# A longitudinal clinical and MRI evaluation of the treatment with erenumab

**DOI:** 10.1007/s10072-020-04660-7

**Published:** 2020-08-26

**Authors:** Alberto Coccia, Caterina Lapucci, Monica Bandettini di Poggio, Matteo Grazzini, Matilde Inglese, Cinzia Finocchi

**Affiliations:** Policlinic San Martino Hospital, Department of Neuroscience, Rehabilitation, Ophthalmology, Genetics, Maternal and Child Health (DINOGMI), University of Genova, Largo Paolo Daneo 3, 16132 Genoa, Italy

Migraine is a very common neurologic disease second only to stroke for number of disability-adjusted life-years and, for the global disease burden [1]. Nowadays, there are several available preventive, abortive, and behavioral therapies for migraine, with different mechanisms of action (MoA).

It has been demonstrated that a neuropeptide (the CGRP—the calcitonin gene–related peptide), along its receptor, located in both central and peripheral neurons can influence neuronal modulation of pain and vascular activity. The role in the pathophysiology of migraine is supported by the localization of the peptide receptors in the dorsal root and trigeminal ganglions. Its involvement in the pathophysiological processes underlying migraine led to the development of CGRP antagonists (the “gepants”) and four different antibodies targeting the CGRP receptor (erenumab) or the CGRP itself (eptinezumab, fremanezumab, and galcanezumab).

Erenumab, a fully human monoclonal antibody directed against the calcitonin gene–related peptide receptor, was consequently approved for the prevention of episodic or chronic migraine. The STRIVE Study **(**A Phase 3, Randomized, Double-blind, Placebo-controlled Study to Evaluate the Efficacy and Safety of AMG 334 in Migraine Prevention) demonstrated that the treatment with erenumab at a monthly dose of 70 mg or 140 mg provides a significant reduction in the frequency of migraine days [2].

The aim of our study is to perform a longitudinal evaluation of the effect of the treatment with erenumab in migraine patients from a clinical and neuroradiological point of view.

Thus, we recruited 22 migraine patients (17 (77%) female and 5 (23%) male) (mean age: 52 years (± 9.8 SD)), who started treatment with erenumab at the dose of 70 mg from May 2019 to December 2020. In 12 (55%), the dose was successively increased to 140 mg.

Follow-up clinical evaluations were scheduled at 3 months and 1 year. At each visit, data about migraine evolution were collected, evaluating monthly number of migraine days, concomitant and acute medications, and Migraine Disability Assessment (MIDAS) questionnaire.

Three-tesla MRI (Prisma, Siemens) was performed in 9 patients at the baseline and scheduled at 3-month and 1-year FU. For each patient and timepoint, MRI protocol included the following sequences: 3D FLAIR (TR: 5000 ms; TE 393 ms; voxel size 0.4 × 0.4 × 1 mm^3^), 3D-MPRAGE (TR: 2300 ms; TE: 3 ms; 1 mm isotropic voxels), T2 space (TR: 3200 ms; TE 564 ms; 1 mm isotropic voxels), PSIR (TR: 5000 ms; TE: 11 ms; voxel size 0.5 × 0.5 × 2 mm^3^), multishell diffusion (TR: 4500 ms; TE: 75 ms; flip angle: 90°; 1.8 mm isotropic voxels), resting state functional MRI (rs-fMRI) (TR: 720 ms; TE: 33 ms; flip angle: 52°; 2.3 mm isotropic voxels), and 3D segmented echo-planar imaging (EPI) providing T2* magnitude and phase contrasts (TR = 64 ms; TE = 35 ms; 0.65 mm isotropic voxels).

For each timepoint, MRI pipeline analysis included the following: (i) white matter (WM) lesion load calculation through manual segmentation by using Jim Xinapse version 7.0; (ii) visual analysis of PSIR and 3D FLAIR images to detect the presence of cortical lesions and their classification in intracortical, leuco-cortical, and subpial; (iii) whole brain, WM and GM volumes estimation by using SPM (CAT12) software; (iv) FLAIR* images generation to detect the presence of the Central Vein Sign (CVS); (v) diffusion analysis, obtained by neurite orientation dispersion and density imaging (NODDI) processing; (vi) Rs-fMRI analysis by using CONN toolbox.

Neuropsychological evaluation, including Simbol Digit Modality Test (SDMT), California Verbal Learning Test (CVLT-II), Brief Visuospatial Memory Test-Revised (BVMT-R), Trail Making Test (TMT A, B), and verbal and phonemic fluency test, was performed at the baseline and scheduled at 3-month and 1-year FU.

Before starting therapy with erenumab, the mean headache day was 18.2 ± 6.7 SD and the mean number of acute medications was 22 ± 8.5 SD.

At 3-month FU, the mean number of migraine days was 11.5 ± 9.9 SD, with a decrease of migraine days at least of 50% in 54% patients (mean reduction: 6.8 ± 4.9 SD days). The number of concomitant medication decreased at 11.6 ± 10.8 SD.

We also observed a reduction of 1.54 grades at MIDAS questionnaire with an average value at baseline of 3.72 and 2.18 at the time of the analysis. In patients with longer FU, benefit was stable over time.

Four patients stopped treatment with erenumab due to lack of efficacy. Common adverse events were constipation and cutaneous rush at the site of injection.

Due to SARS-CoV-2 pandemic, only 2 patients underwent a 3-month MRI FU; for the remaining patients, a 6-month MRI FU has been re-scheduled and it is not yet available.

At baseline, all patients showed WM abnormalities suggestive of migraine on 3D FLAIR images. Fifty-seven WM lesions were globally detected, leading to a mean (SD) lesion volume of 0.31 (0.5) ml. Specifically, 34 (59.6%) were detected in the subcortical WM, 15 (26.3%) in the periventricular areas, and 8 (14%) in the juxtacortical WM. No infra-tentorial WM abnormalities were detected. No cortical lesions were detected by visual inspection of PSIR and 3D FLAIR images.

In conclusion, our analysis confirmed that erenumab is an effective treatment for migraine, resulting in a better quality of life in more than 50% of patients, evaluated through the reduction of migraine days and MIDAS scores. Structural MRI analysis showed findings typical for migraine. The evaluation of structural and functional MRI is ongoing.
